# Associations among chronic obstructive pulmonary disease with asthma, pneumonia, and corticosteroid use in the general population

**DOI:** 10.1371/journal.pone.0229484

**Published:** 2020-02-24

**Authors:** Jun-Jun Yeh, Cheng-Li Lin, Chia-Hung Kao

**Affiliations:** 1 Ditmanson Medical Foundation, Chia-Yi Christian Hospital, Chiayi, Taiwan; 2 Chia Nan University of Pharmacy and Science, Tainan, Taiwan; 3 China Medical University, Taichung, Taiwan; 4 Management Office for Health Data, China Medical University Hospital, Taichung, Taiwan; 5 College of Medicine, China Medical University, Taichung, Taiwan; 6 Center of Augmented Intelligence in Healthcare, China Medical University Hospital, Taichung, Taiwan; 7 Graduate Institute of Biomedical Sciences, College of Medicine, China Medical University, Taichung, Taiwan; 8 Department of Nuclear Medicine and PET Center, China Medical University Hospital, Taichung, Taiwan; 9 Department of Bioinformatics and Medical Engineering, Asia University, Taichung, Taiwan; Srebrnjak Children's Hospital, CROATIA

## Abstract

**Purpose:**

To evaluate the association among chronic obstructive pulmonary disease (COPD) with asthma, steroid use, and pneumonia in the general population.

**Methods:**

Using Taiwan’s National Health Insurance Research Database to identify patients with incident pneumonia, we established a COPD with asthma cohort of 12,538 patients and a COPD cohort of 25,069 patients. In both cohorts, the risk of incident pneumonia was assessed using multivariable Cox proportional hazards models.

**Results:**

The adjusted hazard ratio (aHR) with 95% confidence interval (CI) for incident pneumonia was 2.38 (2.14, 2.66) in the COPD with asthma cohort, regardless of age, sex, comorbidities, and drug use. COPD cohort without inhaled corticosteroids (ICSs) use served as a reference. The aHR (95% CI) for COPD cohort with ICSs use was 1.34 (0.98, 1.83); that for COPD with asthma cohort without ICSs use was 2.46 (2.20, 2.76); and that for COPD with asthma cohort with ICSs use was 2.32 (1.99, 2.72). COPD cohort without oral steroids (OSs) use served as a reference; the aHR (95% CI) for COPD with asthma cohort without OSs use and with OSs use was 3.25 (2.72, 3.89) and 2.38 (2.07, 2.74), respectively.

**Conclusions:**

The COPD with asthma cohort had a higher risk of incident pneumonia, regardless of age, sex, comorbidities, and ICSs or OSs use. COPD cohort with ICSs use did not have a notable risk of incident pneumonia. The COPD with asthma cohort had a higher risk of incident pneumonia, even without ICSs/OSs use.

## Introduction

Pneumonia is a leading cause of death from community-acquired infection. The risk factors for pneumonia are cardiovascular and cerebrovascular diseases, chronic renal diseases, and immunocompromised status (e.g., cancer, steroid use, and old age) [1]. Pneumonia may be considered a systemic inflammatory disorder, and it is characterized by elevated cytokines. Studies have reported elevated cytokine levels in atypical pneumonia (e.g., mycoplasma pneumonia) [2], typical pneumonia (e.g., pneumococcal pneumonia) [3], or other community-acquired pneumonia [4, 5]. In addition, in typical pneumonia (caused by *Pseudomonas aeruginosa*) [6], cytokines regulate the balance between host defense and immunopathology, and they determine the severity of the disease, especially in older people. According to Barens et al. [7, 8], chronic obstructive pulmonary disease (COPD) with asthma cohort is a disorder with components of both asthma and COPD. COPD with asthma cohort has been reported in young adults aged 20 years [9] and in older people [9, 10]. In patients with COPD with asthma cohort, poor lung function is associated with high levels of cytokines (e.g., tissue necrosis factor alpha [TNF-α]) [11]. Moreover, a high level of cytokines other than TNF-α [12] may support the systemic inflammation [9] that occurs in COPD with asthma cohort, which is associated with chronic airway damage in the disease [10, 13].

Studies of the association between COPD with asthma cohort and pneumonia are few, and the effect of inhaled corticosteroids (ICSs) and oral steroids (OSs) use on the development of pneumonia among patients with COPD with asthma cohort has not been addressed [13, 14]. We speculate that typical pneumonia (caused by *Streptococcus pneumoniae*) and atypical pneumonia (caused by *Mycoplasma pneumoniae*) are associated [15–17] with COPD with asthma cohort [16–18]. Therefore, in this study, we examined the relationship between COPD with asthma cohort and incident pneumonia and investigated the effect of ICSs and OSs use on the risk of incident pneumonia in patients with COPD with asthma cohort.

## Methods

### Data source

This retrospective observational cohort study was conducted using the Longitudinal Health Insurance Database 2000 (LHID2000), which is a subset of the Taiwan National Health Insurance Research Database (NHIRD); the LHID2000 is a representative database of 1 million people randomly sampled by the National Health Research Institutes from all enrollees of the National Health Insurance (NHI) program. The details of the NHI program have been well-reported in previous studies [19, 20].

### Ethics statement

The NHIRD encrypts patient personal information to protect privacy and provides researchers with anonymous identification numbers associated with relevant claims information, including sex, date of birth, medical services received, and prescriptions. Therefore, patient consent is not required to access the NHIRD. This study was approved to fulfill the condition for exemption by the Institutional Review Board (IRB) of China Medical University (CMUH104-REC2-115-CR4). The IRB also specifically waived the consent requirement.

### Sampled participants

From the LHID2000, we identified patients aged ≥ 20 years who were diagnosed with COPD (ICD-9 codes 491, 492, and 496) and physician-diagnosed asthma (ICD-9-CM 493) between January 1, 2000, and December 31, 2010, as the COPD with asthma cohort [21–24]. The index date for patients with COPD and asthma (ICD-9-CM code 493) was the date of their first medical visit. We selected patients with COPD and asthma who had at least two outpatient visits or one hospitalization (COPD with asthma cohort). The following exclusion criteria were applied for these patients: missing data for date of birth and sex, age < 20 years, or typical pneumonia diagnosis (ICD-9 codes 481 and 482) and atypical pneumonia diagnosis (ICD-9 codes 483.0 and 483.1) before the index date. From the LHID2000, COPD cohort-control subjects (pure COPD patients) were randomly selected from the remaining COPD patients without COPD with asthma components [25] and atypical pneumonia or typical pneumonia at baseline. The pure COPD patients (COPD cohort) who were frequency matched to each COPD with asthma patient (COPD with asthma cohort) according to age (every 5-year interval), sex, and index year. The index date for COPD cohort was a randomly appointed month and day within the same index year of matched COPD with asthma cohort. The same aforementioned exclusion criteria were applied for COPD cohort.

### Sensitivity analysis

The COPD with asthma cohort and COPD cohort were propensity-score matched at a 1:1 ratio. The propensity score for each patient was calculated using logistic regression, in which the assignment probability was estimated based on baseline variables, including age; sex; comorbidities of diabetes, hypertension, hyperlipidemia, mental disorders, chronic kidney disease, cardiovascular disease (CVD); and ICSs and OSs use. Propensity score matching reduced potential bias. The COPD with asthma cohort and COPD cohort were matched at a 1:1 ratio based on propensity scores. We used logistic regression to calculate the propensity score for each patient by estimating the assignment probability based on baseline variables. This would provide an equal probability to COPD with asthma cohort of being assigned to the COPD cohort (S1 Fig).

### Outcomes and comorbidities

The COPD with asthma cohort, and COPD cohort were followed up until the diagnosis of atypical pneumonia (ICD-9 codes 483.0 and 483.1) or typical pneumonia (ICD-9 codes 481 and 482), loss to follow-up, withdrawal from the NHI program, or December 31, 2011, whichever occurred first. Baseline comorbidities were diabetes, hypertension, hyperlipidemia, mental disorders, chronic kidney disease, and CVD. To avoid immortal time bias, patients diagnosed with COPD with asthma cohort, COPD cohort and those with ICSs new use > 30 days or OSs new use >3 days were also evaluated [26, 27].

### Statistical analysis

The chi-square test and Student’s *t* test were used to examine the differences in categorical and continuous variables, respectively, between the COPD with asthma cohort and COPD cohort. We used the Kaplan–Meier method to estimate the cumulative incidence of atypical pneumonia and typical pneumonia in the COPD with asthma cohort and COPD cohort, and a log-rank test was employed to compare the differences between the two cohorts. The incidence density rates (per 1000 person-years) for atypical and typical pneumonia were calculated for both cohorts and stratified by sex, age group, comorbidity, and ICSs and OSs use. Univariable and multivariable Cox proportional hazards models were used to assess the risks of atypical and typical pneumonia in the COPD with asthma cohort in comparison with the risks in the COPD cohort. The hazard ratio (HR) and 95% confidence interval (CI) were estimated using the Cox models. The Cox models also used to assess the risks of atypical and typical pneumonia in the COPD with asthma cohort in comparison with the risks in the COPD cohort. The multivariable models were adjusted for age; sex; comorbidities of diabetes, hypertension, hyperlipidemia, mental disorders, chronic kidney disease, and CVD; and ICSs and OSs use. The Cox models were also used to estimate HRs and 95% CIs for atypical and typical pneumonia development in COPD with asthma cohort compared with COPD cohort according to propensity score matching. All statistical analyses were performed using SAS 9.4 software (SAS Institute, Cary, NC, USA) for Windows. Two-tailed *P* < 0.05 was considered significant.

## Results

**Fig 1:**
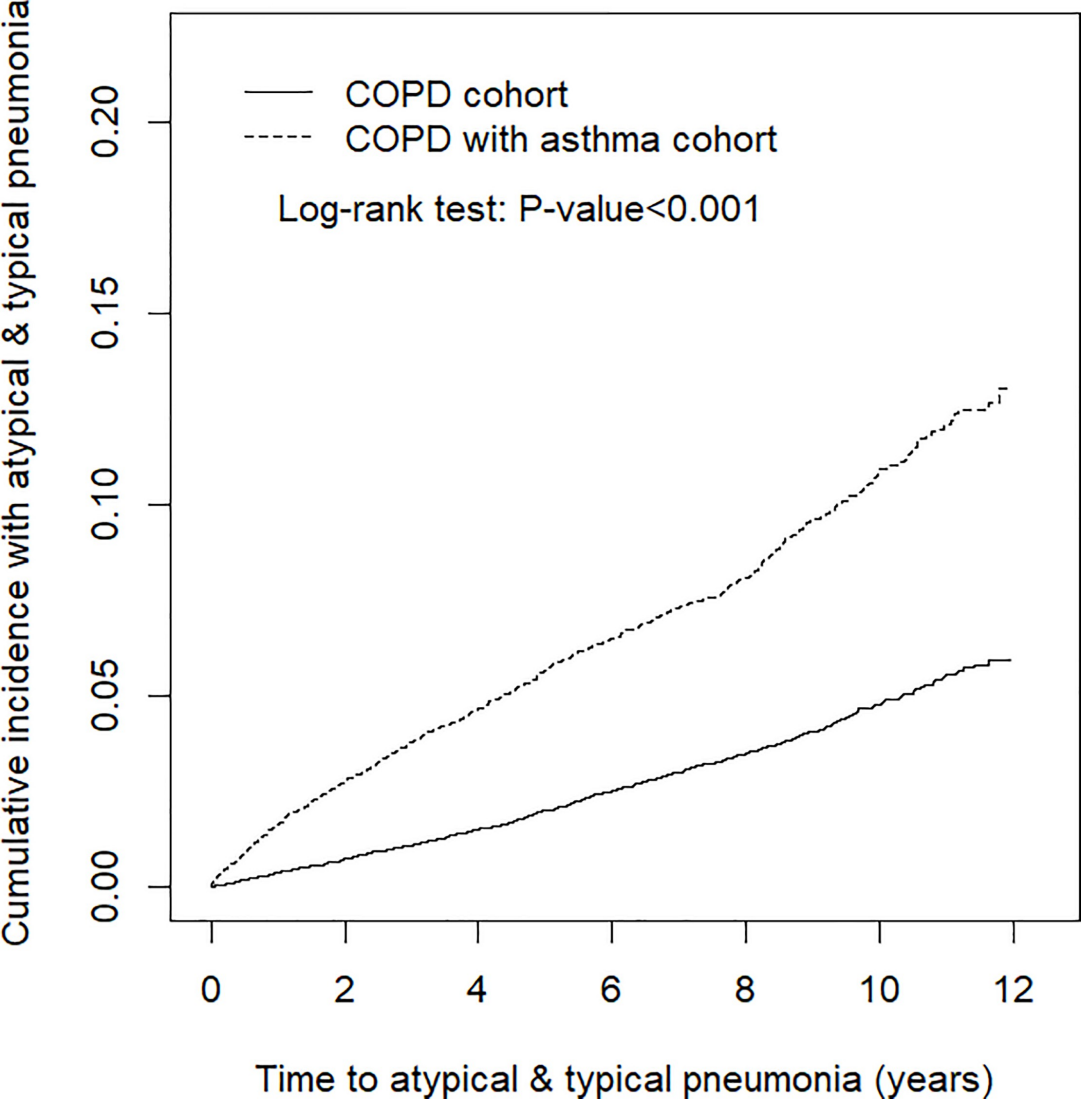
**Cumulative incidence of atypical pneumonia and typical pneumonia in COPD with asthma cohort (dashed line) and COPD cohort (solid line).** COPD, chronic obstructive pulmonary disease.

Data on age, sex, comorbidities, and ICSs and OSs use of the COPD with asthma cohort (N = 12,538) and the COPD cohort (N = 25,069) are shown in Table 1. In both cohorts, most patients were aged ≥ 65 years (55.7%) and were male (56.9%). The mean age was 64.9 ± 14.4 years in the COPD with asthma cohort and 64.7 ± 14.4 years in the COPD cohort. Comorbidities of diabetes, hypertension, hyperlipidemia, mental disorders, and CVD were more prevalent in the COPD with asthma cohort than in the COPD cohort (all *P* < 0.05). In the COPD with asthma cohort matched to COPD cohort the distribution of patient characteristics was similar. Compared with COPD cohort, the COPD with asthma cohort had a higher prevalence of ICSs and OSs use. Specifically, the frequency of ICSs and OSs use was 25.6 and 78.1 in the COPD with asthma cohort and 3.25 and 42.8 in the COPD cohort, respectively. The mean follow-up periods were 6.23 ± 3.42 and 5.54 ± 3.29 years in the COPD with asthma cohort and COPD cohort, respectively. After follow-up for 12 years, the cumulative incidence of atypical pneumonia and typical pneumonia was approximately 7.2% higher in COPD with asthma cohort than in COPD cohort (log-rank test *P* < 0.0001, Fig 1).

**Table 1:** Patient characteristics in the sex-and-age-matched and propensity-score-matched cohorts for the COPD with asthma cohort and COPD cohort.

	Age and Sex Matched					Propensity Score Matched				
Variable	COPD with asthma cohort		COPD cohort			COPD with asthma cohort		COPD cohort		
	(N = 12,538)		(N = 25,069)			(N = 9902)		(N = 9902)		
	n	%	n	%	p-value	n	%	n	%	p-value
**Age, year**					0.99					0.001
20−49	2050	16.4	4100	16.4		1510	15.3	1672	16.9	
50−64	3498	27.9	6996	27.9		3007	30.4	2709	27.4	
≥ 65	6990	55.8	13973	55.7		5385	54.4	5521	55.8	
Mean (SD)^#^	64.9	14.4	64.7	14.4	0.37	64.9	14.7	64.8	13.9	0.01
**Gender**					0.98					0.89
Female	5403	43.1	10805	43.1		4379	44.2	4389	44.3	
Male	7135	56.9	14264	56.9		5523	55.8	5513	55.7	
**Comorbidity**										
Diabetes	1752	14.0	3273	13.1	0.01	1424	14.4	1452	14.7	0.57
Hypertension	7689	61.3	12107	48.3	<0.001	6084	61.4	6119	61.8	0.89
Hyperlipidemia	3832	30.6	6095	24.3	<0.001	3066	31.0	3075	31.1	0.89
Mental disorders	6455	51.5	8491	33.9	<0.001	5080	51.3	5111	51.6	0.66
Chronic kidney disease	336	2.68	654	2.61	0.68	279	2.82	284	2.87	0.83
Cardiovascular disease	6382	50.9	8341	33.3	<0.001	5080	51.3	5111	51.6	0.66
**Medication**										
Inhaled corticosteroids (ICSs)	3204	25.6	814	3.25	<0.001	568	5.74	568	5.74	0.99
Oral steroids (OSs)	9791	78.1	10721	42.8	<0.001	7386	74.6	7377	74.5	0.88

SD, standard deviation.

Chi-square test

^#^*t* test

Table 2 presents the incidence and risks of pneumonia in the COPD with asthma cohort compared to those age-and-sex-matched COPD cohort stratified by sex, age group, and comorbidity. The incidence density rates were 11.5 and 4.57 per 1000 person-years in the COPD with asthma cohort and COPD cohort, respectively (Table 2). According to the multivariable Cox model, the adjusted HR (aHR) for atypical and typical pneumonia development was 2.38 (95% CI = 2.14–2.66) for the COPD with asthma cohort compared with the COPD cohort. In all analyses stratified by sex, age, and comorbidity, the risks of atypical and typical pneumonia were higher in the COPD with asthma cohort than in the COPD cohort.

**Table 2:** Incidence and hazard ratios for atypical and typical pneumonia in the COPD with asthma cohort and COPD cohort stratified by sex, age group, and comorbidity in the age-and-sex-matched cohorts.

	COPD with asthma cohort							
	Yes			No				
Variables	Event	PY	Rate^#^	Event	PY	Rate^#^	Crude HR (95% CI)	Adjusted HR (95% CI)
**All**^1^	900	78132	11.5	749	163946	4.57	2.52(2.29, 2.78)***	2.38(2.14, 2.66)***
**Gender**^2^								
Female	292	35436	8.24	211	73133	2.89	2.86(2.39, 3.41)***	2.60(2.13, 3.17)***
Male	608	42696	14.2	538	90813	5.92	2.40(2.14, 2.70)***	2.28(2.00, 2.60)***
**Age, year**^3^								
20−49	55	15076	3.65	20	30258	0.66	5.53(3.31, 9.22)***	5.65(3.17, 10.1)***
50−64	151	24305	6.21	68	49933	1.36	4.57(3.43, 6.09)***	3.20(2.30, 4.45)***
≥ 65	694	38751	17.9	661	83755	7.89	2.27(2.04, 2.53)***	2.14(1.90, 2.41)***
**Comorbidity**^**§**^^,^^**4**^								
No	96	14115	6.80	109	60373	1.81	3.76(2.86, 4.95)***	3.83(2.77, 5.31)***
Yes	804	64017	12.6	640	103573	6.18	2.03(1.83, 2.25)***	2.33(2.08, 2.61)***

CI, confidence interval; HR, hazard ratio; PY, person-years

Rate^#^, incidence rate, per 1000 person-years

^§^Subjects with any comorbidity of diabetes, hypertension, hyperlipidemia, mental disorders, chronic kidney disease, or cardiovascular disease were classified into the comorbidity group

^1^ Adjusted HR was calculated using Cox proportional hazard regression and was adjusted for age; sex; comorbidities of diabetes, hypertension, hyperlipidemia, mental disorders, chronic kidney disease, and cardiovascular disease; and inhaled corticosteroids (ICSs) and oral steroids (OSs) use

^2^ Adjusted HR was calculated using Cox proportional hazard regression stratified by sex and was adjusted for age; comorbidities of diabetes, hypertension, hyperlipidemia, mental disorders, chronic kidney disease, and cardiovascular disease; and ICSs and OSs use

^3^ Adjusted HR was calculated using Cox proportional hazard regression stratified by sex and was adjusted for age; comorbidities of diabetes, hypertension, hyperlipidemia, mental disorders, chronic kidney disease, and cardiovascular disease; and ICSs and OSs use

^4^ Adjusted HR was calculated by Cox proportional hazard regression stratified by comorbidity and was adjusted for age, sex, and ICSs and OSs use.

***P < 0.001.

Table 3 presents the risks of atypical and typical pneumonia associated with ICS and OSs use by age-and-sex-matched. Compared with COPD cohort without ICSs use, the aHR (95% CI) for COPD cohort with ICSs use was 1.34 (0.98, 1.83); COPD with asthma cohort without ICSs use and with ICSs use had higher risks of atypical and typical pneumonia (aHR = 2.46, 95% CI = 2.20–2.76; aHR = 2.32, 95% CI = 1.99–2.72, respectively). Similarly, compared with COPD cohort without OSs use, aHRs for the risks of atypical and typical pneumonia were 1.16 (95% CI = 1.00–1.34), 3.25 (95% CI = 2.72–3.89), and 2.38 (95% CI = 2.07–2.74) in COPD cohort with OSs use, COPD with asthma cohort without OSs use, and COPD with asthma cohort with OSs use, respectively.

**Table 3:** Adjusted hazard ratios for atypical and typical pneumonia in COPD with asthma cohort with and without ICSs and OSs use during the follow-up period by age-and-sex-matched.

Variables	N	Event	Rate	Crude HR (95% CI)	Adjusted HR^&^ (95% CI)
**COPD cohort**					
Without ICSs	24255	707	4.46	1.00	1.00
With ICSs	814	42	7.93	1.78(1.30, 2.43)***	1.34(0.98, 1.83)
COPD with asthma cohort					
Without ICSs	9334	664	11.8	2.65(2.39, 2.95)***	2.46(2.20, 2.76)***
With ICSs	3204	236	10.7	2.41(2.08, 2.79)***	2.32(1.99, 2.72)***
**COPD cohort**					
Without OSs	14348	342	3.65	1.00	1.00
With OSs	10721	407	5.79	1.59(1.37, 1.83)***	1.16(1.00, 1.34)*
COPD with asthma cohort					
Without OSs	2747	193	12.2	3.35(2.80, 3.99)***	3.25(2.72, 3.89)***
With OSs	9791	707	11.4	3.11(2.73, 3.53)***	2.38(2.07, 2.74)***

COPD with asthma cohort; COPD, chronic obstructive pulmonary disease; rate, incidence rate (per 1000 person-years); ICSs, inhaled corticosteroids; and OSs, oral steroids

^&^Multivariable analysis included age; sex; comorbidities of diabetes, hypertension, hyperlipidemia, mental disorders, chronic kidney disease, and cardiovascular disease; and ICSs and OSs use.

*P < 0.05

***P < 0.001

The risk of atypical pneumonia was 2.96-fold higher in the COPD with asthma cohort than in the COPD cohort by age-and-sex-matched (Table 4). Similar results were observed for typical pneumonia.

**Table 4:** Incidence and hazard ratios for atypical and typical pneumonia in patients with and without COPD with asthma cohort by age-and-sex-matched.

	COPD with asthma cohort							
	Yes			No				
Variables	Event	PY	Rate^#^	Event	PY	Rate^#^	Crude HR (95% CI)	Adjusted HR^&^ (95% CI)
Atypical pneumonia	72	78132	0.92	55	163946	0.34	2.75(1.94, 3.91)***	2.96(1.99, 4.40)***
Typical pneumonia	851	78132	10.9	714	163946	4.36	2.50(2.26, 2.76)***	2.33(2.08, 2.61)***

CI, confidence interval; HR, hazard ratio; PY, person-years

Rate^#^, incidence rate, per 1000 person-years

^&^Multivariable analysis included age; sex; comorbidities of diabetes, hypertension, hyperlipidemia, mental disorders, chronic kidney disease, and cardiovascular disease, and inhaled corticosteroids (ICSs) and oral steroids (OSs) use.

***P < 0.001.

Propensity score matching for sensitive analysis was showed in the Table 5. The incidence density rates for pneumonia were 11.7 and 8.06 per 1000 person-years in the COPD with asthma cohort and the propensity score-matched COPD cohort, respectively The risks of atypical and typical pneumonia were higher in the COPD with asthma cohort than in the COPD cohort (aHR = 1.43, 95% CI = 1.27–1.60, *P* <0.001) (Table 5). The risk of atypical pneumonia in the COPD with asthma cohort was significantly higher by 94% than that in the COPD cohort. The risk of typical pneumonia in the COPD with asthma cohort was significantly higher by 40% than that in the COPD cohort.

**Table 5:** Overall incidence of atypical and typical and pneumonia and estimated hazard ratios in COPD with asthma cohort compared with propensity score-matched COPD cohort.

	COPD with asthma cohort	
	No(COPD, N = 9902)	Yes (N = 9902)
**Pneumonia**		
**Person-years**	62046	60109
**Follow-up time (y), Mean±SD**	6.44±3.26	6.07±3.46
Event, n	500	700
Rate^#^	8.06	11.7
Crude HR (95% CI)	1(Reference)	1.44(1.28, 1.61)***
Adjusted HR^&^ (95% CI)	1(Reference)	1.43(1.27, 1.60)***
**Atypical pneumonia**		
Event, n	32	60
Rate^#^	0.52	1.00
Variables	1(Reference)	1.94(1.26, 2.98)***
Adjusted HR^&^ (95% CI)	1(Reference)	1.94(1.27, 2.99)***
**Typical pneumonia**		
Event, n	477	659
Rate^#^	7.69	11.0
Crude HR (95% CI)	1(Reference)	1.42(1.26, 1.60)***
Adjusted HR^&^ (95% CI)	1(Reference)	1.40(1.25, 1.5)***

CI, confidence interval; HR, hazard ratio; PY, person-years

Rate^#^, incidence rate, per 1000 person-years

^&^Multivariable analysis included age; sex; comorbidities of diabetes, hypertension, hyperlipidemia, mental disorders, chronic kidney disease, and cardiovascular disease; and inhaled corticosteroids (ICSs) and oral steroids (OSs) use.

***P < 0.001.

## Discussion

The main finding of this study is that the COPD with asthma cohort had a higher risk of incident pneumonia, regardless of age, sex, drug use, and comorbidities. Incident pneumonia was associated with young and older age in the COPD with asthma cohort, irrespective of OSs or ICSs use and comorbidities. A prospective study [28] in the United States revealed a high incidence of pneumonia in patients newly diagnosed with asthma and COPD, which is in agreement with our finding. Moreover, a retrospective study comparing a COPD with asthma cohort with a COPD cohort revealed a high risk of incident pneumonia [29], which is consistent with our result. Longitudinal profiling of lung microbiomes in a prospective observational study demonstrated the repeatability of bacterial and eosinophilic COPD exacerbations [30], supporting our result.

Until more definitive studies are conducted, it is reasonable to speculate that clinical history (e.g., frequent wheezing and late-onset asthma) [31], indicators of atopy or allergies (e.g., skin testing and higher IgE) [32], physiology (e.g., spirometry, significant bronchodilator response, and air trapping) [33], imaging (e.g., significant bronchial wall thickening in chest computed tomography [CT]), and biomarkers of inflammation (e.g., higher cytokines and higher blood and sputum eosinophils) can be used for COPD with asthma diagnosis [7, 34]. The higher frequencies of medical service use and ICSs/OSs use, which were 25.6% and 78.1% in the current cohort study, may be associated with higher mortality and morbidity [12, 13] and immunocompromised status such as steroid use [14, 15]. These combined factors may have led to a higher risk of pneumonia in the COPD with asthma cohort than in the COPD cohort. The favorable response to ICSs/OSs in steroid-sensitive patients with COPD with asthma cohort may explain the lower mortality in the COPD with asthma cohort than in the COPD cohort [34]. In a previous study, the prevalence of clinical findings on chest X-ray (CXR) and the pulmonary function test (PFT), the frequency of ICSs/OSs use, and the frequency of medical service use were different between the COPD with asthma cohort and COPD cohort [29]. In the present study, the prevalence of comorbidities and the frequency of drug use were significantly different between the COPD with asthma cohort and COPD cohort (Table 1), supporting previous reports. Therefore, COPD with asthma cohort may be considered another chronic obstructive airway disease [16, 23–26]. In a COPD with asthma cohort study based on the NHIRD, Su et al. reported that ICSs use was 53.48% during follow-up [35]. Shantakumar et al. reported that ICSs and OSs use were 46.1% and 85.5% during 1-year follow-up in the COPD with asthma cohort, respectively [25]. A study of the prevalence of COPD in Taiwan revealed that patients with COPD received examinations such as CXR (84.7%), CT (39.4%), and PFT (58.44%) and were ever-smokers (82.9%) [36]. In our study, the non-ACO cohort was derived from the pure COPD cohort. Moreover, we identified the COPD with asthma cohort based on the ICD code for ICSs/OSs use [25, 32]. These measures may enable the correct identification of patients with COPD with asthma cohort and may avoid indication bias.

ICSs/OSs users have higher health awareness and a healthier lifestyle than do non-ICSs/OSs users [29]. Based on this observation, ICSs/OSs users, especially those with COPD with asthma cohort, are more likely to seek preventive health services including screening tests such as CXR, PFT, sputum culture, and vaccinations [25]. However, measuring lifestyle factors, disease prevention behaviors, and drug compliance in observational studies is difficult. Pneumonia is associated with nutrition and immunocompromised status, including diabetes or hyperlipidemia and steroid use; thus, we included these factors in analysis to avoid health bias. The statistical methodology enables observational studies (e.g., this study) to simulate randomized control trials (RCTs).

Cytokines play a vital role in systemic inflammation and airway infection [37]. A study indicated that the levels of cytokines (e.g., TNF-α) increased in cohorts with asthma and acute exacerbation (AE) of COPD [12]. In addition, high levels of cytokines have been shown to be associated with COPD with asthma cohort [11, 38]. High levels of cytokines contribute to poor lung function [39], which has been previously reported to be a critical factor in pneumonia [40]. Higher cytokine levels in atypical [2] or typical [41] pneumonia support that system inflammation with poor lung function was associated with the pneumonia in the COPD with asthma cohort [5]. Young COPD with asthma cohot [42] without comorbidities may be at a risk of incident pneumonia. Mycoplasma pneumonia is probably a predisposing factor of COPD with asthma cohort, as described in our previous report [17]. The interplay [16] between incident pneumonia and COPD with asthma cohort through cytokines [37] warrants further research.

COPD with asthma cohort comorbidities include smoking-related diseases [43], diabetes, chronic CVD [32], and chronic renal disease, which were associated with incident pneumonia in the present study. In addition, in this study, the COPD with asthma cohort had a higher prevalence of CVDs (50.9%) and hypertension (61.3%), which are associated with congestive heart failure (CHF). In a recent study, Yeh et al. found that CHF is associated with incident pneumonia [44], supporting this result. Older people may exhibit a high risk of incident pneumonia because of the prevalence of CVDs [45] and mental disorders [46] associated with COPD with asthma cohort.

ICSs and OSs are commonly the first choice of treatment for COPD with asthma cohort [47]. Compared with COPD cohort without ICSs use (used as a reference), the risk of incident pneumonia did not significantly increase in COPD cohort with ICSs use. Similar to our result, in a nested case-control study, Mapel et al. revealed that pure COPD cohort with ICSs use did not have a higher risk of incident pneumonia [48]. This critical finding of our study support that the ICSs play an important role of the escalation pathway of the COPD management. An example is that classification D with triple therapy or continue exacerbation need dual or triple therapy including ICSs in the global initiative for chronic obstructive pulmonary disease 2019 (GOLD 2019) [49, 50].

Compared with COPD cohort without OSs use (used as a reference), COPD cohort with OSs use had a higher risk of incident pneumonia. Waljee et al. revealed a high risk of pneumonia in OSs users in a pure COPD cohort [51], which is in accordance with our result.

In the present study, a high risk of incident pneumonia was found in COPD with asthma cohort without or with ICSs/OSs use. In support of this finding, a previous study found a higher risk of incident pneumonia in COPD with asthma cohort with ICSs use than in COPD cohort with ICSs use [29]. A possible explanation is that COPD with asthma cohort are steroid resistant; thus, ICSs do not exert significant effects on improving lung function for this phenotype [7]. Therefore, COPD with asthma cohort with long-term poor lung function may develop incident pneumonia.

The immunosuppressive effects of ICSs on the respiratory epithelium and disruption of the lung microbiome are most likely implicated in the effect of ICSs on the risk of pneumonia [52]. In a US study, patients with pneumonia and community-acquired respiratory distress syndrome (CARDS) toxin exposure [53] exhibited similar histopathological pulmonary changes, suggesting that CARDS toxins play a major role in cross-reaction [16] of the infection–inflammatory response [15, 16]. This supports our results.

A novel finding of this study is the lower risk of incident pneumonia in COPD with asthma cohort with ICSs/OSs use (aHR = 2.32/2.38, respectively) than in COPD with asthma cohort without ICSs/OSs use (aHR = 2.46/3.25, respectively). This finding may imply that COPD with asthma cohort is a different obstructive airway disease. COPD with asthma cohort exhibit lower forced expiratory volume in 1 second/forced vital capacity (FEV1/FVC) [12] and eosinophilic inflammation in the airway [54]. In COPD with asthma cohort, ICSs and OSs may attenuate eosinophilic [53] inflammation in the airway [54] and may have the lower frequency of AE [55], thus improving pulmonary function and quality of life. A favorable longitudinal change in lung function in COPD with asthma cohort with ICSs use is the slower decline of lung function (FEV1/FVC) in these patients compared with COPD cohort [13], supporting our result. Therefore, COPD with asthma cohort receiving optimal doses of ICSs/OSs may have a lower risk of incident pneumonia than COPD with asthma cohort without OSs/ICSs use. However, this finding warrants further research.

As aforementioned, higher prevalence of hypertension and CVDs (e.g., CHF) is associated with incident pneumonia [44]. The higher frequency of AE in COPD with asthma cohort with readmission plays a critical role in the risk of incident pneumonia, especially in patients with late-stage CVDs (e.g., CHF) [56]. Poor lung function is a predictor of readmission [57, 58]. Therefore, the steroid-resistant phenotype in the COPD with asthma cohort may play a role in the risk of incident pneumonia, especially under the vicious cycle of readmission with AE [7].

In this study, the COPD with asthma cohort had a higher risk of incident pneumonia, regardless of ICSs/OSs use, than did the COPD cohort, in accordance with previous reports [29]. Moreover, COPD with asthma cohort with ICSs/OSs use showed a lower risk of incident pneumonia than did the patients without ICSs/OSs use. Based on these findings, physicians should be aware of the high risk of incident pneumonia in the COPD with asthma cohort, thereby enabling physicians to treat incident pneumonia in the early stage in this cohort. Finally, the asthma in young age with poor lung function may develop into the COPD with asthma cohort in previous study [21, 22]. We enrolled the young adult of COPD with asthma cohort aged 20–49 years in this study [23]. This COPD with asthma cohort with young age or old age have higher frequency of medical services in Yeh et al., study [59]. Thus, largely portion of this group patients experienced the OSs usage.

In the LE et al. study suggest that patients treated for severe asthma exacerbation (SAE), the discharge prescription for patients treated for SAE in the emergency room (ER) should at least include a SABA, OSs therapy for a short period, and ICSs therapy if it has not been prescribed before [60]. In 30% of severe adult asthma patients, OSs are required in addition to ICSs to maintain of asthma control [61]. Meanwhile, the OSs have the benefit in the severe COPD or eosinophilic COPD with AE [62, 63]. In addition, the COPD with asthma cohort have the 2 predominant form including the eosinophilic COPD and asthma with smoking history [64, 65]. Therefore, the higher frequency of the acute exacerbation of COPD or asthma in the COPD with asthma cohort receiving the OSs in the ER visit and hospitalization. Chung, et al. found the frequency of the ER visit and hospitalization was 4472 event among 8571 patients having COPD with asthma cohort [29]. Thus, this cohort having the higher frequency of the OSs use. The Shantakumar S et al. study found that 59% COPD with asthma cohort who experienced the exacerbations and 85.5% of these patients having OSs use. These previous reports in line with our result [25, 29]. Furthermore, the chronic asthma with smoking with poor response to ICSs. ICSs were not the initial management role of the COPD cohort such as classification A, B and C in the recommendation of GOLD 2019 [66]. In this study, the COPD with asthma cohort derived from the COPD patients. Thus, the frequency of ICSs use didn’t do a great deal in these COPD subgroups.

These reports and our finding were not against the global initiative for asthma 2019 (GINA 2019) [67]. The ICSs will play a more important role than the OSs especially in mild asthma and in the escalation pathway of the eosinophilic COPD with triple combination-therapy [49]. However, the short course OSs (ex. 5days) in the GINA 2019 was still a critical role in the acute of asthma or COPD with AE in GOLD 2019 in the ER or hospitalization based on the precise medicine [68, 69]. This speculation warrants further research.

### Strength

The findings of studies using the NHIRD are relevant to the general population. The use of the NHIRD in COPD with asthma cohort research has become more popular because an increasing number of scientific reports have been using this database to represent the general population [25]. COPD with asthma cohort codes were confirmed on the basis of drug use, receipt of PFT, imaging studies, and history of high frequency of hospital admission or outpatient visits; therefore, the diagnosis of COPD with asthma cohort is accurate in this database [25, 45, 46]. In this study, we employed age-stratified analysis to avoid competing mortality. The most vital predisposing factors of pneumonia, such as CVD (smoking-related), mental disorders (alcohol-related), immunocompromised diabetes, chronic renal disease, age > 65 years, and OSs/ICSs use, were considered in the analysis. The diagnosis of pneumonia is well established and validated in NHIRD [1, 17, 35].

A care system for chronic diseases has been well established in Taiwan, including telemonitoring in patients with COPD [70]. Lee et al. found that the NHI program is associated with substantial reductions in deaths from infections such as pneumonia [71]. Moreover, the COPD with asthma cohort received more CXR, CT, and PFT examinations than did the pure COPD and pure asthma cohorts in the 12 months from the post-index date, which supports our speculations [25]. These measures may avoid the effects of confounding factors in the follow-up period.

Sensitivity analysis revealed a higher risk of incident pneumonia in the COPD with asthma cohort than in the propensity score-matched COPD cohort. This result validates our primary hypothesis.

### Limitation

This study has some limitations. First, we performed propensity score matching to avoid selection bias. Some authors consider analysis with propensity score matching to be similar to retrospective RCTs; however, this method cannot completely replace RCTs. Second, a dose-dependent (U shape) analysis of drug effects revealed that the drugs may not have an effect on severe deterioration of terminal-stage patients. This may have been another confounding factor in this study [72]. Third, the use of antacid drugs is associated with pneumonia [73], but we did not analyze the effect of this drug. Forth, cytokine data were not retrieved from the NHIRD. Fifth, the COPD patients have the frequency of ever-smoker up to 82.9% in another Taiwan study [36]. We derived this COPD with asthma cohort from the COPD patients, therefore the largely portion of COPD with asthma cohort have the smoking history in this study. However, the pack of cigarettes per day were unavailable in NHIRD. Finally, the data was collected from the date before the December 31, 2011 and cutoff point of age set on the 20 years. The trend of the treatment may change by the time and age such as the change of the GINA 2019. Moreover, we selected the patients with COPD and asthma who had at least two outpatient visits, one hospitalization and receiving new ICSs at least 30 days. Therefore, the patients have received the ICSs before the index date and use days <30 days were not enrolled. Meanwhile, the combined formula such as ICS/ long-acting β_2_-agonist (LABA) was popular in Taiwan [35], we didn’t analyze this formula in this study. This study method may explain to the lower frequency of the ICSs use among COPD with asthma cohort. Thus, apply the policy of the OSs use in the COPD with asthma cohort in future may be the other limitation in our study.

## Conclusion

The COPD with asthma cohort had a higher risk of incident pneumonia, regardless of age, sex, comorbidities, and ICSs or OSs use. The risk of incident pneumonia was higher in young adults and in patients without comorbidities. COPD cohort with ICSs use did not have a significant risk of incident pneumonia. The COPD with asthma cohort had a higher risk of incident pneumonia, even without ICSs/OSs use.

## Supporting information

S1 Checklist STROBE statement—Checklist of items that should be included in reports of observational studies. (DOC)

S1 Fig Numbers of propensity score–matched COPD with asthma cohort, COPD cohort. (DOC)
