# Supplementary material for: Associations among chronic obstructive pulmonary disease with asthma, pneumonia, and corticosteroid use in the general population
Source: PLoS One. 2020 Feb 24;15(2):e0229484. doi: 10.1371/journal.pone.0229484 (PMC7039502; doi:10.1371/journal.pone.0229484)
Supplement: S1 Fig — (DOC) [file pone.0229484.s002.doc]

| COPD with asthma  (N=25069) | Experimental group ( N=9902)  COPD with asthma cohort |
| --- | --- |
| COPD patients  Not meet criteria  (N=12538) | Control group (N=9902)  COPD cohort |

S1 Fig. Numbers of propensity score –matched COPD with asthma cohort , COPD cohort

COPD

Patients
